# Spatiotemporal Quantification of Local Drug Delivery Using MRI

**DOI:** 10.1155/2013/149608

**Published:** 2013-04-24

**Authors:** Morgan B. Giers, Alex C. McLaren, Jonathan D. Plasencia, David Frakes, Ryan McLemore, Michael R. Caplan

**Affiliations:** ^1^Center for Interventional Biomaterials, School of Biological and Health Systems Engineering, Arizona State University, P.O. Box 879709, Tempe, AZ 85287, USA; ^2^Banner Good Samaritan Medical Center, 901 E Willetta Street, 2nd Floor, Phoenix, AZ 85006, USA; ^3^Image Processing Application Laboratory, School of Biological and Health Systems Engineering, Arizona State University, P.O. Box 879709, Tempe, AZ 85287, USA

## Abstract

Controlled release formulations for local, *in vivo* drug delivery are of growing interest to device manufacturers, research scientists, and clinicians; however, most research characterizing controlled release formulations occurs *in vitro* because the spatial and temporal distribution of drug delivery is difficult to measure *in vivo*. In this work, *in vivo* magnetic resonance imaging (MRI) of local drug delivery was performed to visualize and quantify the time resolved distribution of MRI contrast agents. Three-dimensional *T*_1_ maps (generated from *T*_1_-weighted images with varied *T*_*R*_) were processed using noise-reducing filtering. A segmented region of contrast, from a thresholded image, was converted to concentration maps using the equation 1/*T*_1_ = 1/*T*_1,0_ + *R*_1_ *C*, where *T*_1,0_ and *T*_1_ are the precontrast and postcontrast *T*_1_ map values, respectively. In this technique, a uniform estimated value for *T*_1,0_ was used. Error estimations were performed for each step. The practical usefulness of this method was assessed using comparisons between devices located in different locations both with and without contrast. The method using a uniform *T*_1,0_, requiring no registration of pre- and postcontrast image volumes, was compared to a method using either affine or deformation registrations.

## 1. Introduction 

Controlled release formulations for local drug delivery are of growing interest to device manufacturers, research scientists, and clinicians. There are many current and potential applications for controlled release devices, including cancer treatment [1], pain management [2, 3], tissue engineering [4], and infection treatment [5]. For decades, orthopaedic infection management has relied on the use of antimicrobials delivered from bone cement at the infection site [6]. There are an estimated 112,000 total orthopaedic infections of arthroplasties and fracture-fixation devices per year [7], and this number is expected to increase as the projected number of arthroplasties will likely increase by several fold over the next 18 years [8]. Approximately $1.8 billion is spent annually on increased medical costs due to orthopaedic infection of total joint arthroplasties in the USA [7]. Orthopaedic implant infections result from common human skin microbes, such as *Staphylococcus epidermidis* and *Staphylococcus aureus*, and are often complicated by biofilm formation. Biofilm residing microbes are not only protected by transport-limiting polysaccharide matrix, but are more resistant to antimicrobials [9]. Antimicrobial concentration of 100–1000 times the usual minimum inhibitory concentration (MIC) used to treat planktonic microbes are required to treat infections with biofilm effectively [10, 11]. Intravenous delivery to achieve these antimicrobial levels will cause serious systemic toxicity for most of the antimicrobials used to treat implant infections. Local drug delivery at the site of orthopaedic infection is used to achieve effective concentration of antimicrobial without systemic toxicity. 

Even though antimicrobial loaded bone cement (ALBC) is intended for *in vivo *use, most release studies of antimicrobials from ALBC have been performed *in vitro*. For instance, researchers commonly characterize drug elution profiles from controlled release formulations by placing samples of known geometry under near infinite sink conditions, such as a large volume of frequently exchanged fluid [12, 13]. While release studies give valuable information necessary for directly comparing different controlled release formulations, it does not represent how or where the drugs will distribute when the device is implanted. Infinite sink conditions produce the greatest possible release of drug which represents the potential release capability not the actual elution profiles likely to be achieved *in vivo*, where mass transport resistances from the surrounding tissue are likely to decrease the rate of release. *In vivo* studies have been performed [14–17], but none provide comprehensive information on the spatial and temporal distribution of drug delivery. *In vivo* tests frequently focus on efficacy, such as infection control [17], but do not provide details regarding how the antimicrobial is distributed because this is difficult, expensive, and time consuming to measure. *In vivo* animal experiments that do consider spatial distribution of antimicrobial commonly utilize tissue biopsies near implants and collect fluids, such as seroma, blood, and urine [14–16]. These techniques are time consuming to analyze, not comprehensive (e.g., resolution is low due to limited number of samples), and of limited clinical applicability to humans due to their invasiveness and requirements for multiple sampling.

Magnetic resonance imaging (MRI) has been used to visualize distribution of drugs delivered locally in several clinically relevant applications; however, in most of these studies, either concentration of the drug is not calculated or is not defined spatially. Sampson and coworkers deliver MRI contrast agents to brain tumors, but no quantification of the agent's concentration is performed [18, 19]. Krauze et al. [20] and Port et al. [21] imaged liposomal Gd-DTPA delivery, but neither quantified concentration. Fritz-Hansen et al. calculated bulk concentration of contrast in arterial blood, but, was not concerned with spatial distribution [22]. Other studies have considered other aspects of the MRI contrast/concentration relationship based on *in vitro* tests [23–29]. 

Some studies of imaging drug delivery have calculated local concentration of drug, but each has limitations. Sarntinoranont and coworkers have studied delivery of gadolinium diethylenetriaminepentaacetic acid (Gd-DTPA) delivery to brain tumors [30], and they calculate concentration of the Gd-DTPA using a method validated in an agarose phantom [31]. Sarntinoranont et al.'s studies seem to accurately calculate concentration with good spatial resolution; however, in our attempts to utilize a similar method for a different area of the body, additional studies into the potential sources of error and methods to minimize and quantify that error are warranted. Kim et al. quantified the distribution of drugs delivered from an ocular implant using MRI [32]; however, the function used by Kim et al. to convert MR intensity to concentration is similar in shape to a parabola and thus results in two valid concentrations for most MR intensity values—one concentration being high and the other being low; thus, the user must infer which concentration is more likely based on proximity to the depot. Several groups have calculated concentrations of contrast agent *in vitro* [23, 33–36]; however, their methods of quantification are not validated *in vivo* to determine sources of error or to quantify the error likely in their *in vivo* measurements.

In this work, we provide detailed methods for *in vivo* MR imaging of local delivery of Gd-DTPA in an orthopaedic model which provides a rigorous test of the method's ability to distinguish contrast agent from anatomical features, thus, also provides a rigorous test of the method's ability to accurately calculate concentration of the Gd-DTPA. In this model MRI images of Gd-DTPA, delivered from polymethyl methacrylate (PMMA) bone cement, were converted to Gd-DTPA concentration to provide time-resolved maps of Gd-DTPA concentration. The contrast agent, Gd-DTPA, was chosen because of its similar solubility and diffusion coefficient (4.0 × 10^−6^ cm^2^/sec) [37] to the antimicrobials Vancomycin (3.64 × 10^−6^ cm^2^/sec) [38] and Gentamicin (2.08 × 10^−6^ cm^2^/sec) [39], which are common choices to treat infected orthopaedic implants. This paper presents a detailed protocol for performing this method on an animal model. Further, sources of error are discussed and quantified when possible. Finally, methods of image volume registration are demonstrated and compared to the method proposed here (average value of precontrast *T*_1_ applied to all voxels).

## 2. Methods 

### 2.1. Implant Formulation

 PMMA bone cement was formed using Simplex P bone cement (Stryker, Kalamazoo, MI, USA). Control implants, with no contrast agent, were made according to the manufacturer's instructions. Experimental implants were made identically with the addition of either (a) an additional 2.1%v (2.9%w) Gd-DTPA, an MRI contrast agent; 8.8%v (11.4%w) xylitol, a particulate porogen used to increase release rate and amount; and 89.1%v (85.7%w) PMMA and polymerized MMA or (b) an additional 1.1%v (1.4%w) Gd-DTPA, 9.9%v (12.9%w) xylitol, and 89%v (85.7%w) PMMA and polymerized MMA. Implants of all compositions were formed into 3 mm diameter × 7 cm long rods using a red rubber catheter (Covidien, Mansfield, MA, USA) as mold.

### 2.2. Surgical Procedure

 All procedures were compliant with the National Institutes of Health guidelines for the care and use of laboratory animals and approved by the Institutional Animal Care and Use Committee. All studies were performed using New Zealand White rabbits (*n* = 18). Animals were sedated using ketamine (35 mg/kg), xylazine (5 mg/kg), and butorphanol (0.1 mg/kg), and anesthesia was maintained by administering 2% isoflurane during the procedure.

ALBC was implanted in four different ways to study the effect of different sizes/shapes of implants and effect of implant location on distribution of Gd-DTPA from the implant. The rationale for these different implantations and comprehensive discussion of the similarities and differences of resulting Gd-DTPA distribution is published elsewhere [40, 41]. In the first set of procedures, ALBC rods were implanted in either muscle, intramuscular rod (IMR), or the intramedullary canal of the femur, intraosseous rod (IOR) [40]. Briefly, the right quadriceps of each animal received a cement rod of either the experimental (2.1%v Gd-DTPA, 8.8%v xylitol, 89.1%v PMMA, and polymerized MMA) or control (no Gd-DTPA, no xylitol) cement composition. The left femur of each animal received a cement rod of either the experimental (1.1%v Gd-DTPA, 9.9%v xylitol, 89%v PMMA and polymerized MMA) or control composition. In the second set of procedures, either a partial thickness section of muscle (PTM) or a full thickness section of muscle and bone (FTMB) was removed and replaced with bone cement [41]. For the FTMB wound, muscle tissue was removed from the mid quadricep, and a femoral circumference window was created in the anterolateral cortex of the femur. The defect was filled with ALBC (composition as in IMR) or control. In the PTM model, muscle was removed, the dead space was filled with cement of either experimental or control composition (compositions as in IMR).

### 2.3. Image Acquisition

**Figure 1:**
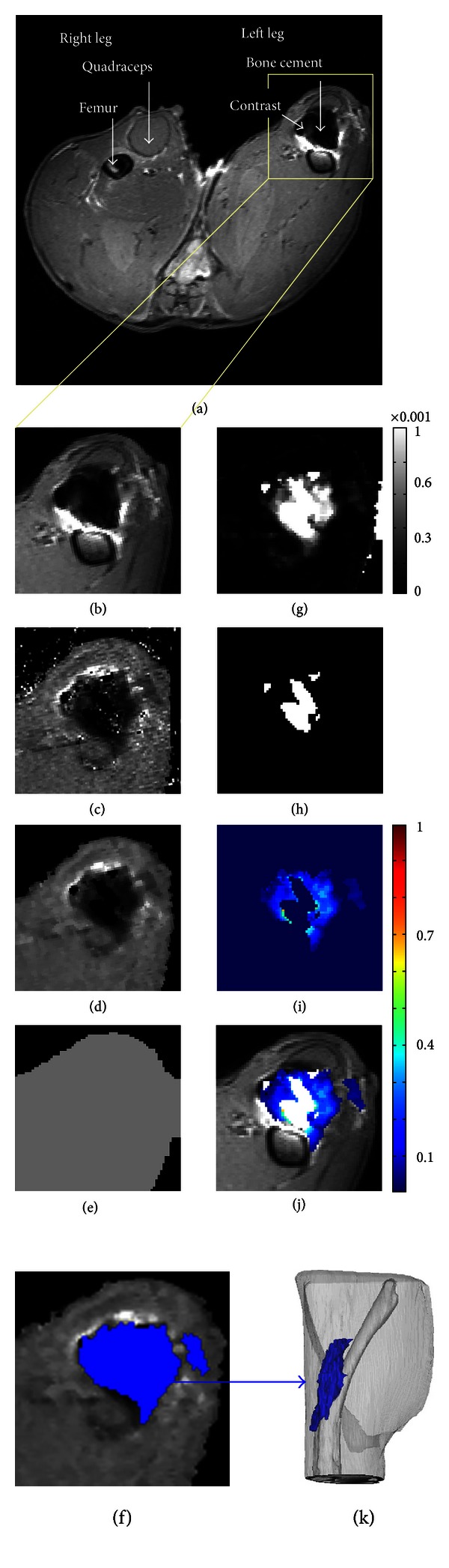
(a) *T*_1_-weighted image, (b) ROI of the *T*_1_-weighted image, (c) original *T*_1_ Map, (d) filtered and masked *T*_1_ map, (e) *T*_1,0_ calculated as one standard deviation less than peak of histogram, (f) segmented region of contrast, (g) 1/*T*_1_ − 1/*T*_1,0_ values plotted, (h) region where *T*_1_ = NA, (i) concentration map where the scale is in mM, (j) concentration map superimposed onto *T*_1_-weighted image, (k) 3D reconstruction from Mimics.

 A series of *T*_1_-weighted rapid acquisition with relaxation enhancement (RARE) scans were taken at repetition times (*T*_*R*_) of 1463, 2000, 3000, and 5000 ms (RARE = 2, no averages) on a Bruker Biospin 7-T MRI (Bruker Biospin, Billerica, MA, USA) every 15 minutes for 4–6 hours (Figure 1(a)). A 15 cm quadrature transceiver coil was used. Flip angle of the RF pulse was calibrated by the Bruker software before each scan, and the images were checked to ensure no ghosting artifacts were present. The images were taken with coronal slices from knee to hip, 42 slices total (field of view = 12 cm), with a voxel size of 0.3 mm × 0.3 mm × 2 mm, where the slice thickness was 2 mm and resultant matrix size was 256 × 256 × 42. This imaging sequence required approximately 14 minutes.

The series of *T*_1_-weighted images at different *T*_*R*_ was used by the Bruker software to construct a longitudinal relaxation time, *T*_1_, map based on the solution to the Bloch equation:

(1) $$\begin{array}{c} S\left(T_{R}\right)=S_{0}\left(1-e^{-T_{R}/T_{1}}\right), \end{array}$$

(2) $$\begin{array}{c} S_{0}=k\rho e^{-T_{E}/T_{2}}, \end{array}$$

where *S* is the signal intensity, *T*_*R*_ is repetition time (time between RF pulses), *T*_1_ is the longitudinal relaxation time, and *S*_0_ is defined by (2), where *k* is the proportionality constant based on instrument factors, *ρ* is the spin density, *T*_*E*_ is the echo time, and *T*_2_ is the transverse relaxation time [42]. The estimated error of this process was calculated by taking the residuals of the curve fitting process for 1 pixel. In a *T*_1_-weighted image, contrast and fat appeared bright, whereas, cement and bone appeared dark as seen in Figure 1(a). In the *T*_1_ map, fat appeared bright, whereas, contrast, bone, and cement appeared dark as seen in Figure 1(b).

### 2.4. Image Processing

 The *T*_1_ maps were imported into MATLAB (Mathworks, Natick, MA, USA). In MATLAB each slice of the *T*_1_ map was separately treated with a noise-reducing filter which changes a pixel to the median value of itself and the 4 in-plane neighbors that share an edge with the pixel. The filtering results are shown in Figure 1(c). Subsequently, a binary mask of the leg area was made by morphologically opening the filtered *T*_1_-weighted image slice, applying a binary threshold, filling holes, and removing groupings of pixels less than 100, then morphologically closing the image slice. The binary mask of the leg area was used to mask noise from outside of the legs in the *T*_1_ map (Figure 1(c)). A histogram of this image was then calculated. The portion of the histogram to the right of the peak (values with *T*_1_ equal to or greater than the peak) was duplicated to the left of the peak to make the histogram symmetric (excluding pixels containing contrast agent) enabling calculation of a standard deviation for determination of a suitable threshold for segmentation. After this, both the *T*_1_-weighted and *T*_1_ map image slices were exported from MATLAB as a series of TIFF files. The TIFF images were imported into Mimics (Materialise, Leuven, Belgium), where the *T*_1_ map was thresholded to a value of *T*_1_ at one standard deviation less than the peak value of the histogram. This threshold level (one standard deviation less than peak of histogram) matched the segmentation performed by several expert users.

We then employed a semiautomatic gradient flow detection algorithm in Mimics to create the 3D representation of areas containing Gd-DTPA. First, all pixels within the muscle tissue of the leg were segmented from the thresholded region. Then, all the pixels connected to the implant in this region were segmented. This gave a region of all the pixels connected to the implant within the muscle of the leg, which included the cement implant and contrast agent. After muscle implants and contrast were segmented, several steps were performed to segment contrast within the intramedullary canal of the femur. The intramedullary canals of both femurs (both legs) were segmented from the *T*_1_-weighted image volume using a semiautomatic gradient flow detection algorithm, which is similar to the method shown by Karasev et al. [43]. The mask of the segmented region from the femur without contrast (one of two legs contained a PTM experiment which does not contain ALBC in the intramedullary space) was imported into MATLAB, where a symmetric histogram was created for the intramedullary space as described above. The peak value and standard deviation of the symmetric histogram were calculated. In Mimics, the masked intramedullary space of the femur containing an implant and contrast agent was thresholded to *T*_1_ one standard deviation less the peak value of the histogram. All pixels connected to the cement implant within this thresholded region were segmented.

Additionally, to provide anatomical reference in the 3D images, the exterior of the cortex of the femur was segmented using a semiautomatic gradient flow detection algorithm in Mimics, manual correction, and a 3D object smoothing function. The legs were also segmented for an anatomical reference using thresholding, manual correction, and 3D object smoothing. The segmented femur, legs, and contrast were plotted together as 3D objects using Mimics as shown in Figure 1(h).

The segmented regions of contrast and cement were exported as a series of mask images in a bitmap format. The bitmaps were imported back into MATLAB where they were transformed into a binary image mask. The binary image mask was multiplied by the *T*_1_ map to give a map in only the area of contrast (Figure 1(d)). This region was transformed into a concentration map (Figures 1(e) and 1(f)) using the following:

(3) $$\begin{array}{c} \frac{1}{T_{1}}=\frac{1}{T_{1,0}}+r_{1}C, \end{array}$$

(see [28, 31, 33]), where *T*_1,0_ is a precontrast *T*_1_ map value and *T*_1_ is the postcontrast *T*_1_ map value. For (3), the peak value from the histogram for the appropriate tissue (muscle or intramedullary canal) was used for *T*_1,0_. Relaxivity (*r*_1_) of the contrast agent was set to 0.0038 mM^−1^ s^−1^ which is consistent with reported values from several literature studies using 3–7T MRIs, including Rohrer et al. who obtained this particular value for Gd-DTPA in serum using a 4.7T MRI [27]. The concentration map was superimposed onto a *T*_1_-weighted image to provide the anatomical details as shown in Figure 1(g).

To evaluate the validity of the use of a single *T*_1,0_ value rather than a pixel-by-pixel *T*_1,0_ value achieved by image registration, histograms of 5 precontrast rabbits (rabbits no. 1, 8, 9, 15, 18 in the series) were composed. We then used the standard deviations from these histograms to perform sensitivity analysis on the concentrations calculated for different *T*_1_ values using the peak *T*_1,0_ value with *T*_1,0_ one standard deviation greater than and less than the mean *T*_1,0_. To evaluate if one *T*_1,0_ value could be used for multiple tissue types, histograms were calculated for the femur and the muscle separately. We also compare/contrast the accuracy of the above technique with using *T*_1_ values from an image in which no contrast is present. This requires that the image volumes with no contrast be spatially registered to the image volumes with contrast present. Such a registration was completed for one image set. First, a 3D rigid body affine registration was performed in which matching points on the femur in precontrast and postcontrast images were chosen by a user. A transformation matrix was created and optimized using singular value decomposition similar to a method outlined by Eggert et al. [44]. The precontrast image volume was transformed using a 3D linear interpolation algorithm, which used Delaunay triangulation to handle the scattered data points. Then a 3D deformation registration was performed by picking points from the affine registered precontrast image and postcontrast image. A transformation map was generated by calculating the difference between current and desired point location for the points chosen, then interpolating all the surrounding pixel values using linear interpolation. The image volume was transformed using the same linear interpolation algorithm as in the affine registration.

Although varying concentration of the contrast agent does effect the magnetic susceptibility and thus the relaxivity of the contrast agent, (3) seems to accurately calculate concentration using a constant value of *r*_1_ (0.0038 mM^−1^ s^−1^) between approximately 100 *μ*M and 5 mM (shown in Figure 3). Data in Figure 3 were acquired by scanning a series of vials containing known concentrations of Gd-DTPA in 2%w agarose, plotting 1/*T*_1_ versus concentration, and comparing to (3) (dashed line).

### 2.5. Image Analysis

 Volumes of segmented contrast, including the cement implant, were calculated. These were adjusted by subtracting the volume of cement implanted, as calculated from the weight of the implant (see details of the surgical insertion). The volumes of the region where *T*_1_ = NA, which includes the cement implant and a region of extremely high concentrations of contrast (>50 mM), were calculated. Total mass of contrast agent was calculated by summing all concentrations from pixels with a real *T*_1_ value and multiplying by voxel volume (0.18 *μ*L).

Volumes and total mass were analyzed for significance by two-way ANOVA (wound types and presence of contrast agent: experimental IMR, experimental IOR, control IMR, and control IOR) using Minitab (Minitab Inc., State College, PA, USA). Post hoc *t*-tests were performed when *P* < 0.05 by ANOVA.

## 3. Results and Discussion

**Figure 2:**
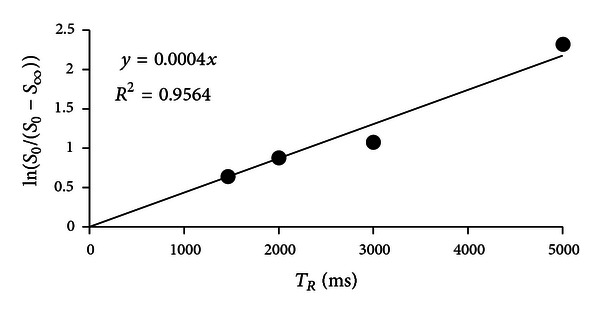
A plot of signal intensity from the *T*_1_-weighted images at different *T*_*R*_, for one pixel, used to determine *T*_1_ value.

Equations (1) and (2) were used to calculate *T*_1_ from the intensity values from a set of *T*_1_-weighted images taken at different relaxation times (*T*_*R*_). The fitting is performed pixel-by-pixel. There is noise in the *T*_1_-weighted images; thus, there is noise in the *T*_1_ value obtained. The noise in the *T*_1_ values depends on the signal-to-noise ratio (SNR) of the image acquisition method used. ln⁡(*S*_0_/(*S*_0_ − *S*_*∞*_)) is plotted versus *T*_*R*_ for a single pixel in the muscle of specimen 4 (Figure 2); the inverse of the slope is the *T*_1_ value, and an estimate of the error can be determined from the residuals (Figure 2). This algorithm results in *T*_1_ < 1 ms for some pixels, and these pixels are set to *T*_1_ = NA (not applicable). For the pixel in Figure 2, the *T*_1_ value is 2500 ms, and the residuals squared are 0.96, indicating that a good fit is achieved. Noise is visible as graininess in the *T*_1_ map image (Figure 1(b)). Including more values of *T*_*R*_ decreases the error and improves the calculation, but this requires longer image acquisition time. The time required for the scan is also a function of the *T*_*R*_ values chosen, number of slices, and resolution desired. For the 4 *T*_*R*_ values used here (1463, 2000, 3000, and 5000 ms), 42 slices and 0.3 mm × 0.3 mm resolution, a scan takes 14 minutes. Certain applications, such as imaging a beating heart, require a fast measurement time. In those cases, a 14-minute scan is unacceptable so a single *T*_1_-weighted image can be used in such cases [22, 33, 35]. These methods typically result in greater error, but the error can be offset by acquiring a greater number of replicates. 

Next, the *T*_1_ map is filtered to decrease noise (Figure 1(c)). Filtering increases confidence that voxels included as containing contrast are not a product of noise, but filtering also reduces the ability to detect small features in the image. In order for a voxel to be included as having contrast, at least two neighboring pixels must also have contrast. Consequently, a single voxel that contains contrast will be changed to the median value of the surrounding pixels, thus losing the information in the voxel containing contrast. Other sharp features such as tissue planes and bone edges can be replaced (if less than one voxel thick) or thinned by the filtering technique. The order of the filtering technique affects the severity of these changes, with higher order (including more neighboring voxels) making the effects more severe. Here, a 5th-order filtering method is applied (pixel + 4 in-plane neighbors), and this seems to remove much of the noise while only losing very fine features in the image.

Next, the pixels containing contrast agent are identified (Figures 1(e) and 1(f)). In previous work by these authors, blinded reviewers chose areas of contrast from image slices thresholded at 1400 ms, and there was good agreement among reviewers (intraclass correlation coefficient = 0.92–0.96) [40]. In the present work, the method was made even more robust by thresholding at a level based on the longitudinal relaxation times within a single tissue (muscle or intramedullary canal), and including all voxels with *T*_1_ less than the threshold using a semiautomated gradient flow detection algorithm employed in Mimics.

**Figure 3:**
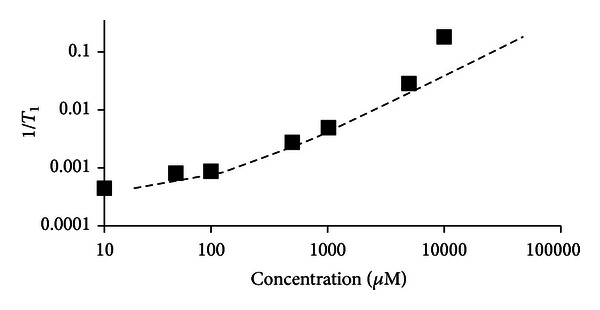
*T*_1_ maps were acquired for a series of different concentrations of Gd-DTPA prepared in agarose gel. The plot shows the difference between actual concentration (squares) and concentration calculated using (3) (dashed line).

**Figure 4:**
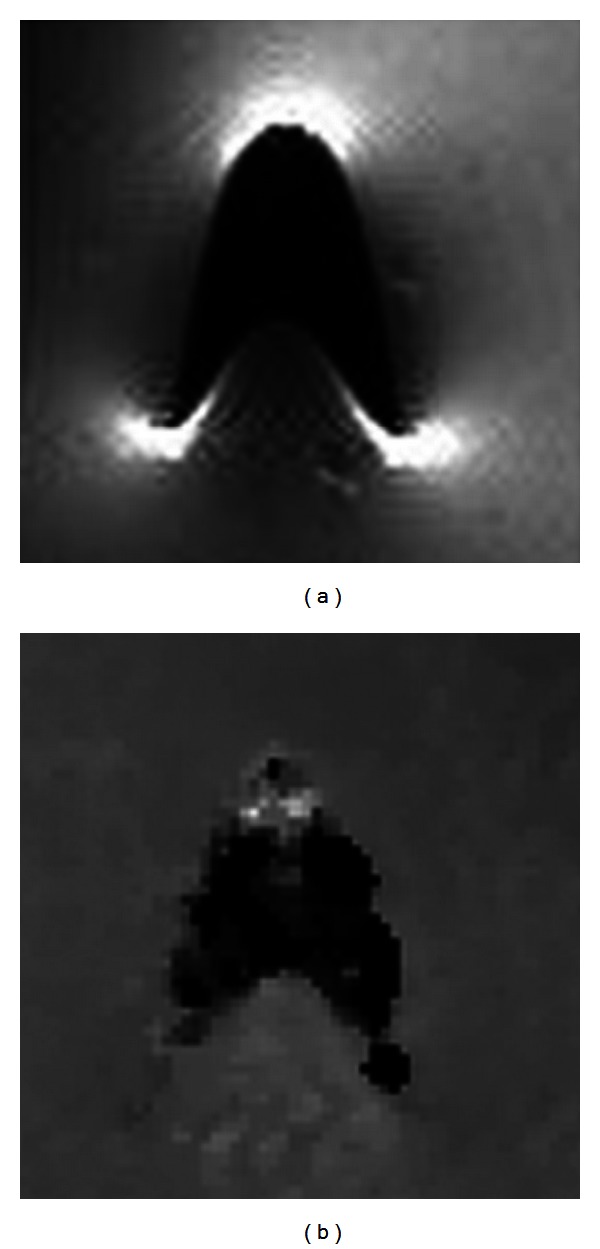
(a) *T*_1_-weighted image and (b) *T*_1_ map of a vial of 100 mM Gd-DTPA which creates an artifact. The dark portion of the images should be round, and the dark portion of the *T*_1_-weighted image should be bright.

Concentrations were calculated by applying (3) to each pixel containing contrast agent. Pixels with a *T*_1_ = NA are excluded from this calculation and assumed to either contain high concentration of Gd-DTPA or be voxels containing cement which has a very low water content. Equation (3) relates *T*_1_ with contrast concentration, but it is only accurate within a range of concentrations (100 *μ*M to 5 mM). Within this range, (3) is not exact because the relaxivity (*r*_1_) can vary depending on local variation of magnetic field strength, molecular microenvironment, binding to macromolecules, access to intracellular or extracellular water, and water exchange rates [28]. Thus, although Figure 3 shows that plotting 1/*T*_1_ versus concentration of samples with known concentrations of Gd-DTPA matches (3) well, it is possible that *r*_1_ values *in vivo* (where more variation in microenvironment is likely) may vary more and cause error in the calculation of Gd-DTPA concentration using (3). At low concentrations, which produce *T*_1_ values close to native tissue, the likely error between the calculated and actual concentrations is fairly large (Figure 3); however, the error is skewed so that the actual concentration is not likely to be much greater than the calculated value, but the actual concentration may be substantially less than the calculated value. When Gd-DTPA exceeds approximately 10–50 mM, an artifact occurs due to the transition of the material properties from paramagnetic to superparamagnetic. This change affects the ability of the MRI to encode spatial information through frequency encoding [42]. This results in *T*_1_ = NA not only in a pixel containing contrast greater than this concentration but also in some nearby pixels due to this error in spatial encoding as shown in Figure 4. The range of concentration between these high (leading to artifact) and low (100 *μ*M) values should be considered when choosing the amount of contrast agent to load into the drug delivery vehicle. The concentration of Gd-DTPA loaded into the ALBC in this study (67 mM) is great enough to allow for an artifact to occur. Most images are unaffected because the Gd-DTPA in the ALBC is not near water and, once it is released into the volume surrounding the ALBC, it quickly becomes diluted to less than the concentration causing artifacts; however, in some images, high concentrations near the femur cause spatial morphing indicating an artifact. The magnitude of this effect was estimated by comparing the volume of pixels where *T*_1_ = NA between control and experimental implants. If there were a significant amount of artifact or superthreshold gadolinium near the implant in the images with contrast, the volume of pixels where *T*_1_ = NA would be higher than in the control images. From the ANOVA, there is no statistically significant differences between the control and experimental (*P* = 0.86), indicating that artifacts present are not large enough to significantly affect the experiment and that the *T*_1_ = NA pixels are most likely pixels containing cement which has a very low water content. The possibility of artifacts must be balanced against the necessity for visualization of contrast agent further away from the implant when choosing the Gd-DTPA loading amount. 

**Figure 5:**
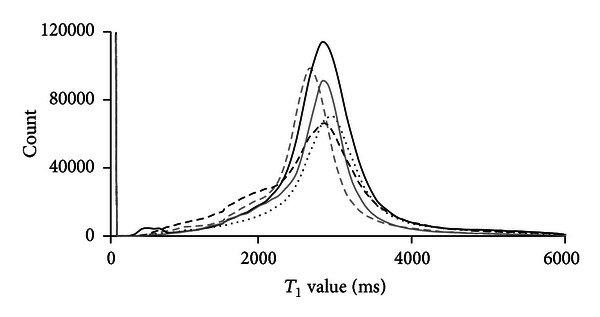
Histograms of 5 precontrast rabbit *T*_1_ maps: rabbit 1 (dashed grey line), rabbit 2 (solid grey line), rabbit 3 (dotted black line), rabbit 4 (dashed black line), and rabbit 5 (solid black line).

**Figure 6:**
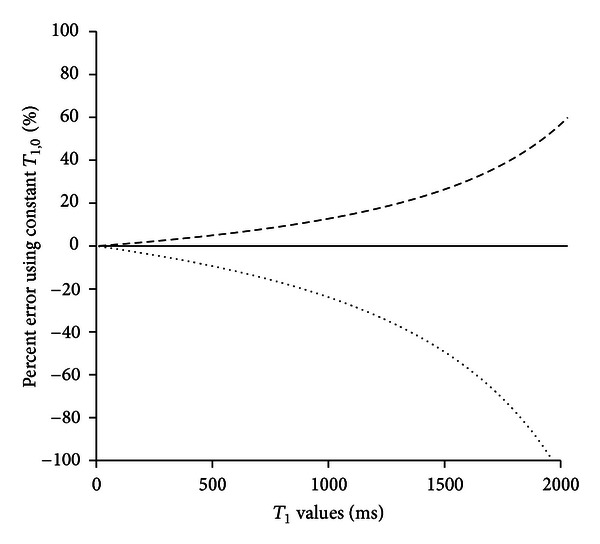
Estimation of sensitivity of concentration calculated using isotropic *T*_1,0_ = 2817 ms (mean of Figure 5 histograms, solid line), 3669 ms (one standard deviation greater, dashed line), and 1965 ms (one standard deviation less, dotted line).

The histograms of five precontrast image volumes were analyzed to find the mean *T*_1_ value of tissue containing no contrast agent (2817 ± 852 ms) (Figure 5) for use on image volumes for which no precontrast image was taken, thus avoiding the need for image registration or using a unique value for each animal. This value, 2817 ± 852 ms, is based on both muscle and intramedullary canal tissue, and the analyses shown in Figures 5 and 6 are based on these unsegregated *T*_1,0_ values. Our current technique for image analysis uses two different *T*_1,0_ values: one for tissue outside of the femur and a separate *T*_1,0_ for the intramedullary canal. The analyses in Figures 5 and 6 provide a quantitative estimate of the error of using one value of *T*_1,0_ to calculate concentration in an entire region of pixels based on the histogram of that tissue's precontrast *T*_1_ values. Using tissue-specific *T*_1_ values (e.g., for muscle and intramedullary canal) decreases the error in each tissue. The mean (2817 ± 852 ms) provided similar information to the histogram peak values (largest count number in the histogram) for the 5 rabbits shown (2815 ± 132 ms). Animal-to-animal variability can be assessed by comparing a single histogram's mean and standard deviation (2905 ± 834 ms) to the mean from the compounded 5-rabbit histogram (2817 ± 852 ms), whose deviation overlaps considerably. Despite the fact that *T*_1,0_ values can vary with metabolic activity, the animal-to-animal variability is small relative to the spread of the histogram. Thus error from animal-to-animal variability is less than error due to differences within a single animal. This indicates that there is minimal error introduced by using the 2817 ms value for all animals rather than using a value determined for each animal.

To quantify error likely resulting from using an average value of *T*_1,0_ rather than a registered precontrast image volume to provide a pixel-by-pixel value of *T*_1,0_, we applied (2) to *T*_1_ values between 0 and 1965 using *T*_1,0_ = 2817 ms (mean), 3669 ms (+1 standard deviation), and 1965 ms (−1 standard deviation) (Figure 6). This provides a reasonable estimate of the effect that large variability in observed *T*_1_ would have on the calculation of concentration. Equation (2) applied to *T*_1_ = 1650 ms results in a concentration of 66 ± 22/40 *μ*M (where the first error number is the difference calculated using *T*_1,0_ = 3669 ms and the second number is the difference calculated using *T*_1,0_ = 1965 ms). As can be seen in Figure 6, error becomes less as *T*_1_ decreases (actual concentration increases). Note that the error is unequal above and below the concentration. For *T*_1_ = 1965 ms, using *T*_1,0_ = 3669 ms calculates a concentration value 54% greater than that calculated using *T*_1,0_ = 2817 ms, whereas using *T*_1,0_ = 1965 ms calculates a concentration of 0 *μ*M (100% error). The error is always greater for lower concentrations. At low values of *T*_1_ (high concentrations), the error is minimal. For example, *T*_1,0_ = 51.5 ms results in a concentration of 5000 ± 20/40 *μ*M (0.4%/0.8%).

**Figure 7:**
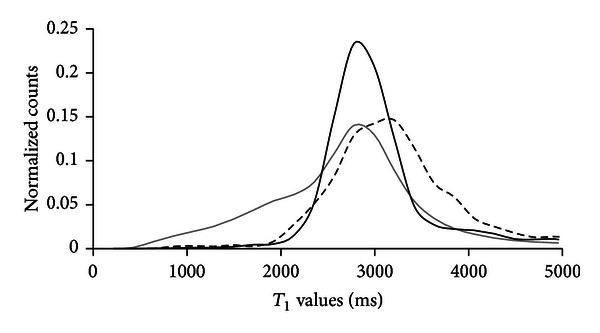
Histograms of the total image (solid grey line), the muscle tissue (solid black line), and the bone (dashed black line) in a precontrast *T*_1_ map.

The uneven error results in concentrations that are more likely to be overestimated rather than underestimated. In other words, a pixel calculated to contain 41 *μ*M contrast agent (*T*_1_ = 1965 ms) may contain no contrast agent at all, but it is unlikely to contain any more than 62 *μ*M. Also, if a pixel has a *T*_1_ value greater than the threshold (1965 ms) (thus is calculated to have no contrast agent present), it is unlikely to have concentration greater than 62 *μ*M. The concentration calculation error will be greater in some areas than in others. For example, the femur has a broader histographic distribution than the total image, as shown in Figure 7, so in the femur, error will be greater than the previous estimate. The muscle is more isotropic than the total image so the error for calculations performed in muscle will be slightly less than the previous estimate. Therefore, using an isotropic *T*_1,0_ values can give accurate order of magnitude information, but specific values, especially low concentration values, should be considered with caution. One potential clinical application of this technique is codelivering Gd-DTPA with antimicrobials to determine if the infection is being treated effectively. For this application, the minimum effective concentration of antimicrobial is near the lower limit of detection of the isotropic *T*_1,0_ technique (20–200 *μ*M). At that lower limit, if a pixel shows as containing contrast (*T*_1_ ≤ 1965 ms), it may or not contain effective concentration of antimicrobial; however, if a pixel does not show as containing contrast (*T*_1_ > 1965 ms), then it likely contains less than an effective concentration of antimicrobial. Therefore, it is unlikely that a patient would receive an additional intervention unnecessarily, but a patient requiring additional intervention could be evaluated to require no additional intervention allowing a risk that the infection could recur.

**Figure 8:**
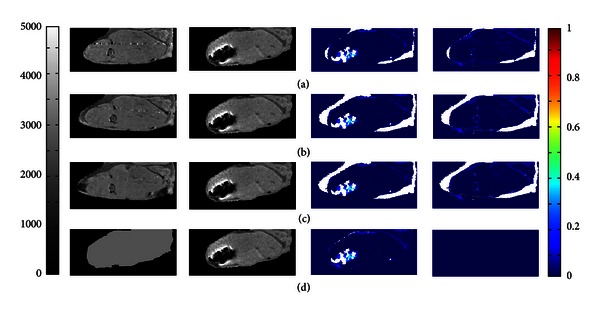
(a) The unregistered images, (b) affine registered images, (c) registration with deformation, (d) constant *T*_1,0_, where the left image is *T*_1,0_, 2nd column image is *T*_1_, the 3rd column image is the concentration map resulting from those *T*_1,0_ and *T*_1_ images, and the right image is the difference between concentration maps for (a), (b), or (c) with (d). White represents regions in which *T*_1_ = NA or for which concentration calculate is out of the range of the color bar (0 mM-1 mM).

Next we compare and contrast results when a single isotropic value of *T*_1,0_ is used (as described above) versus when *T*_1,0_ values are taken from image volumes of the tissue prior to the addition of contrast agent. Images of a precontrast and postcontrast FTMB procedure are shown in Figure 8. Figure 8(a) (left) shows a precontrast image that has not been altered; Figure 8(b) (left) shows the same image but registered to the postcontrast image using an affine registration (rigid body registration); Figure 8(c) (left) shows the same image but registered to the postcontrast image using a deformation registration; and, finally, Figure 8(d) (left) shows the isotropic *T*_1,0_ method in which a single value of *T*_1,0_ is applied to all of the pixels in the region of interest. It is apparent in Figures 8(a)–8(c) (right) that the edges of the legs do not perfectly overlap (large red region in concentration map) in the unregistered, affine registered, or deformation registered images, but the isotropic *T*_1,0_ concentration map (Figure 8(d), right) does not have significant patches of red surrounding the leg indicating that this is not a problem for the isotropic *T*_1,0_ method. The rigid body transformation (Figure 8(b)) was performed by choosing points on the femur, which is a rigid anatomical feature. While the transformation worked well for the femur, the surrounding soft tissue is not registered using this technique. The registration with deformation was applied to register the soft tissue (Figure 8(c)); however, several factors made the registration with deformation method less capable of describing the transform well. It was difficult to identify landmarks to register by in the muscle tissue and especially the fat marrow tissue. Furthermore, choosing the number of corresponding points necessary to obtain a better transform in 3D would be impractically time consuming (250 points takes ~4 hours). Even though the registration with deformation was not perfect, it seems to perform better than the isotropic *T*_1,0_ method for some anatomic features having *T*_1,0_ values different from the tissue mean. For example, in Figure 8(d) (right), fairly thick features appearing to have nonzero contrast agent concentration appear. These features also appear in the registered concentration maps (Figures 8(b), right and 8(c), right), but the features are generally fewer and thinner. This indicates that, for anatomical locations such as the brain, which is less isotropic than the muscle, registration may be more necessary and practical. The brain is simpler to register because of the lack of deformation and multiple landmarks to register by. There are many groups working on performing and automating registration techniques that could be useful if registration were required [45–50]. Regardless of the strengths and weaknesses of each method, within the region likely containing contrast agent (bottom left corner of the leg), all four methods seem to perform well, and no major differences are noted among the methods. There are slight differences in the concentrations calculated in the isotropic *T*_1,0_ method near the edge of the leg; however, these differences are not likely to affect conclusions drawn from these data since animal-to-animal variability is likely greater than error due to the value of *T*_1,0_ used. It should be noted that even though the registration and isotropic *T*_1,0_ methods give similar results for this application, the isotropic *T*_1,0_ method is far less time consuming and has the practical benefit of not requiring a precontrast image (which requires that the animal be scanned, removed from the scanner, and then implanted with the local drug delivery vehicle). For applications where the precontrast and postcontrast image could be obtained without removing the subject from the MRI, such as when the contrast or delivery vehicle is injected, the precontrast image could easily be used for *T*_1,0_ without needing to perform a registration. Therefore, the practicality of a method for a specific anatomical region and the expected performance of a method for that anatomical region should be considered when choosing whether to use a registration technique or an isotropic *T*_1,0_ method.

**Figure 9:**
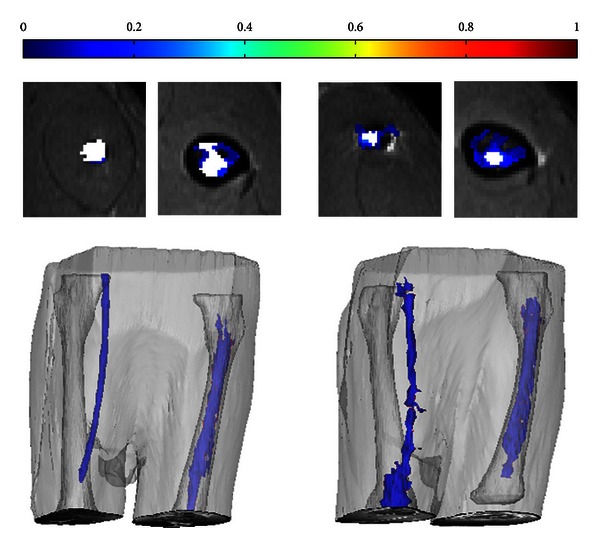
A comparison of concentration maps and 3D Mimics reconstructions of an IMR and IOR without (left) and with (right) contrast agent mixed into the ALBC. Color bar for concentration maps is from 0 mM to 1 mM; white represents region where *T*_1_ = NA.

Figure 9 shows concentration maps and 3D reconstructions for an IMR and IOR of the control and experimental cement composition. Visual examination of the sagittal concentration maps from the dataset shows contrast above and below the IMR. The isotropic *T*_1,0_ contrast concentration calculation method calculates a significant difference in volume of distribution between control and experimental animals with an IMR (*P* < 0.0001) (Figure 9); however, no significant difference is found between control and experimental IOR (*P* ≈ 0.5). When the same implants were compared with total mass of contrast agent observed as the metric, the IMR again showed significance (*P* < 0.005) and the IOR showed no significance (*P* ≈ 0.8). This likely indicates that, in the femur, it is more difficult to distinguish between pixels above the threshold containing contrast and not containing contrast. This is likely due to the broader distribution of precontrast *T*_1_ values (*T*_1,0_) in the intramedullary canal; thus, the error in calculating concentrations in this region is greater.

## 4. Conclusions 

This paper demonstrates a simple to use method for imaging local drug delivery and calculating its local concentration with good spatial and temporal resolution. This method has broad applications in the field of drug delivery, but here is shown applied to delivery from ALBC for the treatment and prevention of infection in orthopaedic applications. We identify and quantify sources of error in this method and suggest ways to minimize these errors. Specifically, we discuss how to generate images with *T*_1_ values in the range that will yield accurate concentrations and avoid artifacts from excessive concentration of contrast agent, the strengths and weaknesses of several methods of generating *T*_1,0_ values for use in converting from *T*_1_ to concentration, and methods for using these data to statistically compare contrast agent distributions between wound models.

## References

[1] Wolinsky JB, Colson YL, Grinstaff MW (2012). Local drug delivery strategies for cancer treatment: gels, nanoparticles, polymeric films, rods, and wafers. *Journal of Controlled Release*.

[2] Coluzzi F, Mattia C (2010). OROS hydromorphone in chronic pain management: when drug delivery technology matches clinical needs. *Minerva Anestesiologica*.

[3] Hurlbert RJ, Theodore N, Drabier JB, Magwood AM, Sonntag VKH (1999). A prospective randomized double-blind controlled trial to evaluate the efficacy of an analgesic epidural paste following lumbar decompressive surgery. *Journal of Neurosurgery*.

[4] Ladewig K (2011). Drug delivery in soft tissue engineering. *Expert Opinion on Drug Delivery*.

[5] Dunbar MJ (2009). Antibiotic bone cements: their use in routine primary total joint arthroplasty is justified. *Orthopedics*.

[6] Buchholz HW, Elson RA, Engelbrecht E, Lodenkämper H, Röttger J, Siegel A (1981). Management of deep infection of total hip replacement. *Journal of Bone and Joint Surgery. British*.

[7] Darouiche RO (2004). Treatment of infections associated with surgical implants. *The New England Journal of Medicine*.

[8] Kurtz S, Ong K, Lau E, Mowat F, Halpern M (2007). Projections of primary and revision hip and knee arthroplasty in the United States from 2005 to 2030. *Journal of Bone and Joint Surgery. American*.

[9] Stewart PS, Costerton JW (2001). Antibiotic resistance of bacteria in biofilms. *The Lancet*.

[10] Ceri H, Olson ME, Stremick C, Read RR, Morck D, Buret A (1999). The Calgary Biofilm Device: new technology for rapid determination of antibiotic susceptibilities of bacterial biofilms. *Journal of Clinical Microbiology*.

[11] Diefenbeck M, Mückley T, Hofmann GO (2006). Prophylaxis and treatment of implant-related infections by local application of antibiotics. *Injury*.

[12] Klekamp J, Dawson JM, Haas DW, DeBoer D, Christie M (1999). The use of vancomycin and tobramycin in acrylic bone cement: biomechanical effects and elution kinetics for use in joint arthroplasty. *Journal of Arthroplasty*.

[13] McLaren AC, Nugent M, Economopoulos K, Kaul H, Vernon BL, McLemore R (2009). Hand-mixed and premixed antibiotic-loaded bone cement have similar homogeneity. *Clinical Orthopaedics and Related Research*.

[14] Nijhof MW, Dhert WJA, Tilman PBJ, Verbout AJ, Fleer A (1997). Release of tobramycin from tobramycin-containing bone cement in bone and serum of rabbits. *Journal of Materials Science: Materials in Medicine*.

[15] Sterling GJ, Crawford S, Potter JH, Koerbin G, Crawford R (2003). The pharmacokinetics of Simplex-tobramycin bone cement. *Journal of Bone and Joint Surgery. British*.

[16] Adams K, Couch L, Cierny G, Calhoun J, Mader JT (1992). In vitro and in vivo evaluation of antibiotic diffusion from antibiotic- impregnated polymethylmethacrylate beads. *Clinical Orthopaedics and Related Research*.

[17] Cierny G, DiPasquale D (2002). Periprosthetic total joint infections: staging, treatment, and outcomes. *Clinical Orthopaedics and Related Research*.

[18] Raghavan R, Brady ML, Rodríguez-Ponce MI, Hartlep A, Pedain C, Sampson JH (2006). Convection-enhanced delivery of therapeutics for brain disease, and its optimization. *Neurosurgical Focus*.

[19] Sampson JH, Raghavan R, Brady ML (2007). Clinical utility of a patient-specific algorithm for simulating intracerebral drug infusions. *Neuro-Oncology*.

[20] Krauze MT, Forsayeth J, Park JW, Bankiewicz KS (2006). Successful and safe perfusion of the primate brainstem: in vivo magnetic resonance imaging of macromolecular distribution during infusion. *Pharmaceutical Research*.

[21] Port RE, Schuster C, Port CR, Bachert P (2006). Simultaneous sustained release of fludarabine monophosphate and Gd-DTPA from an interstitial liposome depot in rats: potential for indirect monitoring of drug release by magnetic resonance imaging. *Cancer Chemotherapy and Pharmacology*.

[22] Fritz-Hansen T, Rostrup E, Larsson HBW, Søndergaard L, Ring P, Henriksen O (1996). Measurement of the arterial concentration of Gd-DTPA using MRI: a step toward quantitative perfusion imaging. *Magnetic Resonance in Medicine*.

[23] Donahue KM, Burstein D, Manning WJ, Gray ML (1994). Studies of Gd-DTPA relaxivity and proton exchange rates in tissue. *Magnetic Resonance in Medicine*.

[24] Fleckenstein JL, Canby RC, Parkey RW, Peshock RM (1988). Acute effects of exercise on MR imaging of skeletal muscle in normal volunteers. *American Journal of Roentgenology*.

[25] Noordin S, Winalski CS, Shortkroff S, Mulkern RV (2010). Factors affecting paramagnetic contrast enhancement in synovial fluid: effects of electrolytes, protein concentrations, and temperature on water proton relaxivities from Mn ions and Gd chelated contrast agents. *Osteoarthritis and Cartilage*.

[26] Prantner AM, Sharma V, Garbow JR, Piwnica-Worms D (2003). Synthesis and characterization of a Gd-DOTA-D-permeation peptide for magnetic resonance relaxation enhancement of intracellular targets. *Molecular Imaging*.

[27] Rohrer M, Bauer H, Mintorovitch J, Requardt M, Weinmann HJ (2005). Comparison of magnetic properties of MRI contrast media solutions at different magnetic field strengths. *Investigative Radiology*.

[28] Stanisz GJ, Henkelman RM (2000). Gd-DTPA relaxivity depends on macromolecular content. *Magnetic Resonance in Medicine*.

[29] Strich G, Hagan PL, Gerber KH, Stutsky RA (1985). Tissue distribution and magnetic resonance spin lattice relaxation effects of gadolinium-DTPA. *Radiology*.

[30] Magdoom KN, Pishko GL, Kim JH, Sarntinoranont M (2012). Evaluation of a voxelized model based on DCE-MRI for tracer transport in tumor. *Journal of Biomechanical Engineering*.

[31] Chen X, Astary GW, Sepulveda H, Mareci TH, Sarntinoranont M (2008). Quantitative assessment of macromolecular concentration during direct infusion into an agarose hydrogel phantom using contrast-enhanced MRI. *Magnetic Resonance Imaging*.

[32] Kim H, Robinson MR, Lizak MJ (2004). Controlled drug release from an ocular implant: an evaluation using dynamic three-dimensional magnetic resonance imaging. *Investigative Ophthalmology and Visual Science*.

[33] Xu F, Han H, Zhang H, Pi J, Fu Y (2011). Quantification of Gd-DTPA concentration in neuroimaging using T1 3D MP-RAGE sequence at 3.0 T. *Magnetic Resonance Imaging*.

[34] Hittmair K, Gomiscek G, Langenberger K, Recht M, Imhof H, Kramer J (1994). Method for the quantitative assessment of contrast agent uptake in dynamic contrast-enhanced MRI. *Magnetic Resonance in Medicine*.

[35] Bokacheva L, Rusinek H, Chen Q (2007). Quantitative determination of Gd-DTPA concentration in T 1-weighted MR renography studies. *Magnetic Resonance in Medicine*.

[36] Mørkenborg J, Pedersen M, Jensen FT, Stødkilde-Jørgensen H, Djurhuus JC, Frøkiær J (2003). Quantitative assessment of Gd-DTPA contrast agent from signal enhancement: an in-vitro study. *Magnetic Resonance Imaging*.

[37] Foy BD, Blake J (2001). Diffusion of paramagnetically labeled proteins in cartilage: enhancement of the 1-D NMR imaging technique. *Journal of Magnetic Resonance*.

[38] Bleicher K, Lin M, Shapiro MJ, Wareing JR (1998). Diffusion edited NMR: screening compound mixtures by affinity NMR to detect binding ligands to vancomycin. *Journal of Organic Chemistry*.

[39] Gordon CA, Hodges NA, Marriott C (1991). Use of slime dispersants to promote antibiotic penetration through the extracellular polysaccharide of mucoid *Pseudomonas aeruginosa*. *Antimicrobial Agents and Chemotherapy*.

[40] Giers MB, Estes CS, McLaren AC, Caplan MR, McLemore R (2012). Jeannette Wilkins Award: can locally delivered gadolinium be visualized on MRI? A pilot study. *Clinical Orthopaedics and Related Research*.

[41] Giers MB, McLaren AC, Schmidt KJ, Caplan MR, McLemore R Magnetic resonance imaging drug distribution following local delivery in surgical wounds.

[42] Haacke EM, Brown RW, Thompson MR, Venkatesan R (1999). *Magnetic Resonance Imaging: Physical Principles and Sequence Design*.

[43] Karasev P, Kolesov I, Chudy K, Tannenbaum A, Muller G, Xerogeanes J Interactive MRI segmentation with controlled active vision.

[44] Eggert DW, Lorusso A, Fisher RB (1997). Estimating 3-D rigid body transformations: a comparison of four major algorithms. *Machine Vision and Applications*.

[45] Zhuang X, Arridge S, Hawkes DJ, Ourselin S (2011). A nonrigid registration framework using spatially encoded mutual information and free form deformations. *IEEE Transactions on Medical Imaging*.

[46] Boehler T, Zoehrer F, Harz M, Hahn HK (2012). Breast image registration and deformation modeling. *Critical Reviews in Biomedical Engineering*.

[47] Loeckx D, Slagmolen P, Maes F, Vandermeulen D, Suetens P (2010). Nonrigid image registration using conditional mutual information. *IEEE Transactions on Medical Imaging*.

[48] Rueckert D, Sonoda LI, Hayes C, Hill DL, Leach MO, Hawkes DJ (1999). Nonrigid registration using free-form deformations: application to breast MR images. *IEEE Transactions on Medical Imaging*.

[49] Zhe L, Deng D, Guang-Zhi W (2012). Accuracy validation for medical image registration algorithms: a review. *Chinese Medical Sciences Journal*.

[50] Frakes DH, Dasi LP, Pekkan K (2008). A new method for registration-based medical image interpolation. *IEEE Transactions on Medical Imaging*.

